# Purification, characterization and functional site prediction of the vaccinia-related kinase 2A small transmembrane domain

**DOI:** 10.1016/j.mex.2022.101704

**Published:** 2022-04-16

**Authors:** Rashmi Puja, Ayon Chakraborty, Shubhankar Dutta, Kakoli Bose

**Affiliations:** a Integrated Biophysics and Structural Biology Lab, ACTREC, Tata Memorial Centre, Tata Memorial Centre, Sector 22, Kharghar, Navi Mumbai 410210, India; b BARC Training School Complex, Homi Bhabha National Institute, Anushaktinagar, Mumbai 400094, India

**Keywords:** Small protein purification, Transmembrane domain, VRK2A, TAK1, Molecular docking, Protein-protein interaction

## Abstract

Vaccinia-related kinases (VRK) are serine-threonine kinases that regulate several signaling pathways. The isoform-VRK2A of one such kinase VRK2 controls cell stress response by interacting with TAK1, a mitogen-activated protein 3 kinase (MAP3K), via its partly cytosolic C-terminal transmembrane domain (VTMD). To establish the driving force and identify the key residues of the VRK2A-TAK1 interaction, we expressed and purified the standalone 3.6 kDa VTMD in the bacterial system using a unique and atypical two-step approach, when the effort to obtain full-length VRK2A remained unsuccessful. Characterization of biophysical properties demonstrated that VTMD domain maintains its structural integrity. Furthermore, dissecting the VRK2A-TAK1 binding interface using *in silico* tools provided important cues toward engineering the VRK2A-TAK1 interface to modulate its functions with desired characteristics. Most importantly, this novel purification strategy demonstrates its universal applicability in protein biochemistry research by serving as a model system for obtaining difficult-to-purify small proteins or domains.

• VRK2A is a highly disordered transmembrane (TM) kinase, whose TM domain interacts with TAK1 (transforming growth factor-β-activated kinase).
• The standalone VRK2A-TM domain (VTMD) was purified using affinity chromatography followed by two-step centricon based approach.
• Biophysical and *in silico* analyses confirmed structural integrity of the domain.

## Graphical abstract

**Figure:**
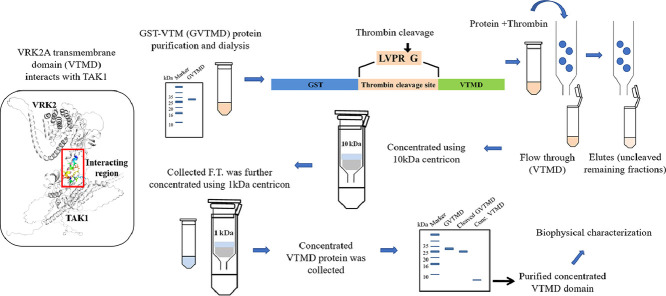

Specifications table

**Table:** 

Subject Area:	Biochemistry, Genetics and Molecular Biology
More specific subject area:	Small membrane domain purification
Method name:	Two step novel protein purification of small transmembrane domain and biophysical characterization
Name and reference of original method:	GST Affinity chromatography Harper S, Speicher DW. Purification of proteins fused to glutathione S-transferase. Methods Mol Biol. 2011;681:259-280. doi:10.1007/978-1-60761-913-0_14 The aforementioned article represents a general principle for GST tagged protein purification. However, novelties of our work lies in purifying small 3 kDa transmembrane protein through two step approach.
Resource availability:	*Not applicable*

## Background

Physiological processes are predominantly regulated by a wide array of proteins involved in multiple signaling pathways. Kinases and phosphatases that modulate protein functions through their phosphorylation/dephosphorylation determine the smooth functioning of signaling pathways. Kinases coordinate several fundamental biological functions including cell cycle progression, apoptosis, and metabolism and hence, they constitute important therapeutic targets. Although kinase domains have been the popular targets for designing inhibitors against associated diseases, the futility of many such endeavors can be attributed to overlooking the contributions from the other essential domains of the kinase proteins. Furthermore, the design of specific inhibitors is often limited by cross-reactivity due to the conserved nature of the catalytic domain leading to undesirable toxicity and side effects [1]. Thus, studying kinase structure and functions using an integrative approach might open up new avenues toward successful drug discovery.

One such family of kinases is vaccinia related kinase family of proteins (VRK). VRKs constitute a diverse class in human kinome [2]. The uniqueness of this family of proteins is manifested by its evolutionary divergence with increasing complexity of the species suggesting the requirement of distinct functional homologs in higher organisms [3]. Therefore, while *C.elegans* and other invertebrates have a single VRK protein, human VRK family comprises three members (VRK1-3) [3].

Human VRKs are a group of serine/threonine kinases that are involved in several critical physiological functions such as regulation of apoptosis, cell cycle, and autophagy through their involvement in a wide spectrum of signaling pathways and hence are clinically important [3,4]. Interestingly, only VRK1 and VRK2 are catalytically active thus suggesting that they have evolved to perform discrete cellular functions. Furthermore, these proteins demonstrate low sequential and structural similarity and are regulated by distinct protein-protein interactions; many are yet to be identified.

VRK2 (vaccinia related kinase 2), the most intriguing and multifaceted kinase of the family has two isoforms (VRK2A and VRK2B), generated due to alternative splicing. VRK2A, the longer version with 508 amino acids, is tethered to the membranes of endoplasmic reticulum and mitochondria via its C-terminal tail that includes a transmembrane domain [5]. On the other hand, VRK2B (1-397 residues) that lacks the C-terminal region is found to be present both in the nucleus and the cytoplasm and is restricted to a few cell types including cancers such as adenocarcinomas [6]. Both the isoforms have a similar site for substrate phosphorylation and sensitivity toward kinase inhibitors [7]. VRK2A, a ubiquitously expressed protein, is an effector of multiple signaling pathways such as mitogen-activated protein (MAP) kinase, ERK1/2 and AKT that regulate apoptosis, autophagy and tumor cell growth [4,8]. It is also implicated to negatively regulate several cancers including breast and adenocarcinoma [6,8].

Despite its important physiological functions and involvement in several diseases, structural and biophysical characterization of full-length VRK2A is not available except for the structural information of inhibitor-bound ΔC-terminal region. Allosteric modulation of VRK2A functions as well as protein-protein interactions involving the C-terminal region is critical towards its association with many pathophysiological functions. For example, MAPKsignaling pathways are known to bemodulatedthrough its transmembrane domain (residues 480-508) tethered to the endoplasmic reticulum in breast-cancer cells [9]. Furthermore, although literature suggests interaction with and subsequent regulation of TAK1 (transforming growth factor β-activated kinase 1), a hypoxia-induced MAP kinase kinase kinase (MAPKKK) by the C-terminal region of VRK2A [9], further quantitative characterization of the interaction has not been possible due to the lack of a purified VTMD (VRK2A Transmembrane Domain). Small protein domains are often purified with large tags such as GST (Glutathione S-transferase) and MBP (Maltose binding protein). Although these tags have their own advantages, they often interfere with the domain structure and function thus creating a bottleneck in such research endeavors [10]. Therefore, understanding the structure and functional properties of this standalone domain is important for specifically targeting VRK2A.

This manuscript aims at filling in the gap by purifying the small C-terminal region of the protein. Using novel biochemical and biophysical approach, we have purified VTMD to ∼ 95% purity and characterized the protein domain. VTMD was found to be reasonably stable as a standalone protein domain. Furthermore, for the first time we have identified the TAK1 interaction site on VRK2A using *in silico* tools. This study thus will provide possibilities for distinctively modulating VRK2A functions with desired characteristics.

## Method details

**I. PredictingVRK2A-TAK1 interacting site and *in silico* identification of the minimal binding region**

1. **Molecular docking analysis of VRK2A and TAK1**
  ➢ Due to the absence of crystallographic information for full-length VRK2A and TAK1 proteins, their AlphaFold predicted models (VRK2: AF-Q86Y07-F1 and TAK1: AF-O43318-F1) were retrieved and subjected to blind docking.
  ➢ Blind docking was conducted using Bioluminate interface (Schrödinger, LLC, New York, NY, 2020) that uses PIPER protein-protein docking program [11,12].
  ➢ A total of 45583 structures were generated that were clustered into 120 docked poses based on several parameters such as electrostatic, hydrophobic and van der Waals interactions were generated and ranked according to the cluster size [13].
  ➢ Top five docked poses representing the top five largest clusters were further subjected to Prime MM-GBSA (Molecular mechanics using Generalized Born and surface area continuum) docking for binding energy and k_d_ value calculation of the complexes (Prime, Schrödinger, LLC, New York, NY, 2020) [14].
  ➢ The complex with the best docking score was chosen for interaction analysis using PDBsum and later given for MD simulation analysis [15].
2. **Molecular Dynamics (MD) Simulation**
  ➢ The top-ranked complex was subjected to MD simulation using Desmond (Desmond, Schrödinger, LLC, New York, NY, 2020) where OPLS3e (Optimized Potentials for Liquid Simulations Version 3e) force field was used to generate topology and parameter files [16,17].
  ➢ The protein structure was surrounded by a cubic box of TIP3P water molecules with the nearest distance from the protein to the box boundary being no more than 10 Å, followed by neutralization [18]. The system was energy-minimized using conjugated gradient for 5000 picosecond (ps) [19].
  ➢ The system was equilibrated in NVT (constant number of particles, volume, and temperature) and NPT (constant number of particles, pressure, and temperature) ensembles with two sets of restrained NVT (for 24 ps and 2000 ps, respectively) and one set of restrained (for 24 ps) and unrestrained (for 5000 ps) NPT each [20].
  ➢ During equilibration, LINCS (LINear Constraint Solver) constraint algorithm was used to apply position restraining force on all the atomic bonds present in the systems [21].
  ➢ Final MD simulation run was executed for 200 nanoseconds (ns) under no-restrained NPT ensemble where the resultant trajectories were used to generate clusters at 10 picosecond intervals.
  ➢ A total of 20,000 structures were generated and accumulated in a cluster to assess the interacting H-bond interactions.
  ➢ The interacting residues identified by the PDBsum server were assessed at each structure in the clusters, and the percentage survival index or PSI (percentage ratio between number of interaction occurrences and total number of cluster structures) was calculated.

**Fig. 1:**
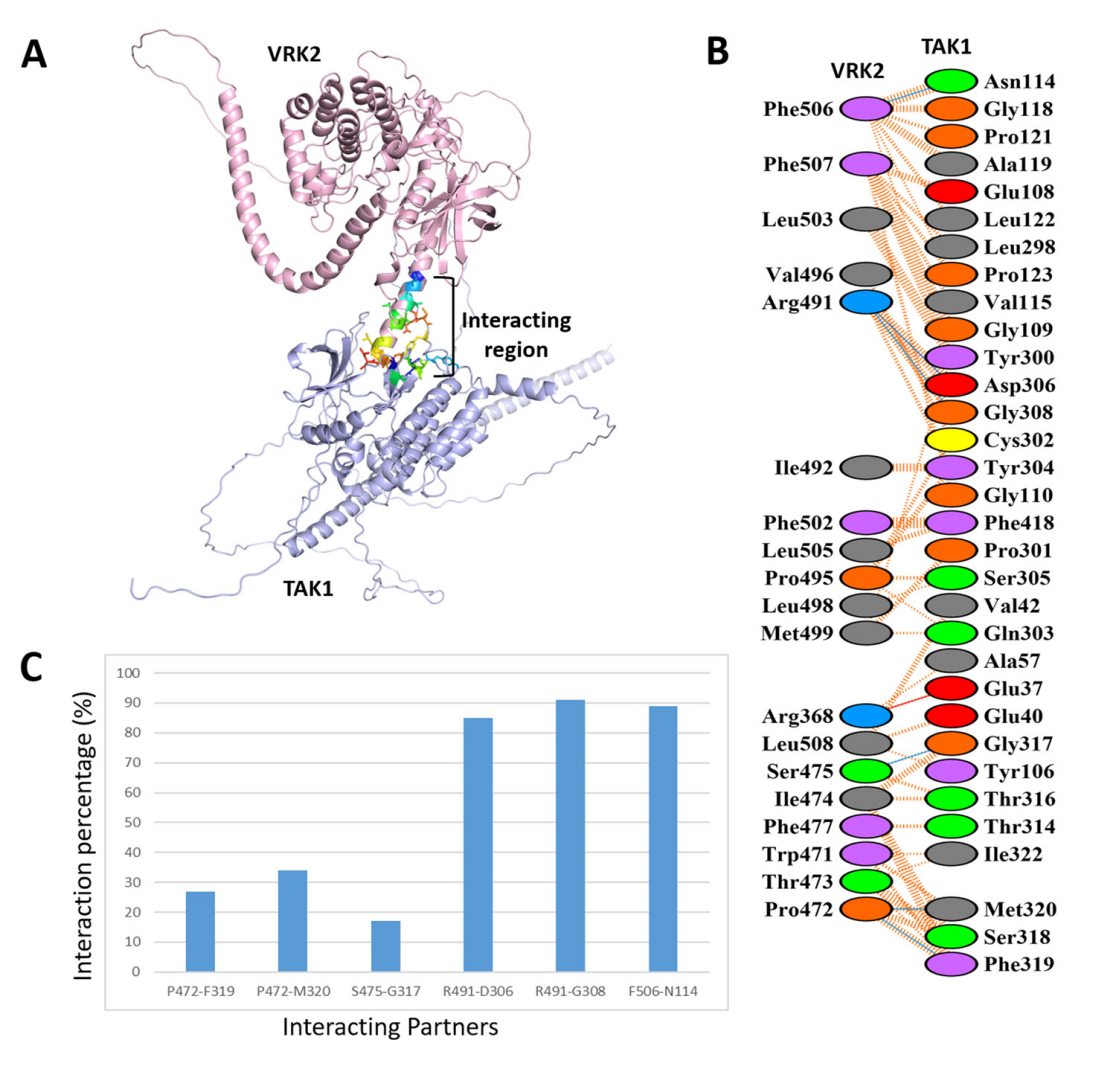
*Interaction analysis of VRK2A and TAK1*. **(A)** Cartoon representation of the VRK2A (light pink)-TAK1 (light blue) top-ranked complex, showing interacting region in rainbow-coloured sticks. **(B)** PDBSum details of their interaction where H-bonds, salt-bridges and non-bonded contacts are denoted as blue, red and orange dashes, respectively. **(C)** Graphical representation of the H-bond interaction percentage calculated over the clusters for 200 ns.

Earlier, the interacting surface between VRK2A and TAK1 [9] was not well-defined. To delineate this, blind docking of VRK2A and TAK1 AlphaFold models resulted five docking clusters with scores or ΔG values spanning -18.2 and-10.9 kcal/mol (**Table S1**). The RMSD (Root Mean Squared Deviation) calculation of these clusters determined the stability of these poses. The complex with the highest docking score and lowest RMSD (**Table S1**) was taken for PDBSum analysis, which showed VTMD residues were interacting with TAK1. The VTMD domain was chosen for subsequent biophysical analyses. Although VTMD has been implicated in interaction with TAK1 [9], the critical residues involved in this interaction were not identified. PDBSum analysis of the top-ranked VTMD-TAK1 complex demonstrated a total of six H-bonds, two salt-bridges and 146 non-bonded contacts. VTMD residues involved in the H-bond interactions were P472, S475, R491 and F506, out of which P472 and R491 form two H-bonds with TAK1 residues (Fig. 1**A** and **B**). To further understand the strength of these H-bonds MD simulation of the complex was carried on for 200 ns that generated a total of 20,000 structures. Interaction analysis of these resultant structures showed that out of 20,000, 18197 (∼91%) structures had H-bond between R491 (VRK2A) and G308 (TAK-1) intact (Fig. 1**C**). Apart from that, 17793 (∼89%), 16991 (∼85%), 6803 (∼34%), 5407 (∼27%) and 3394 (∼17%) structures also preserved the H-bonds of F506-N114, R491-D306, P472-M320, P472-F319 and S475-G317, respectively. Besides forming two H-bonds, R491 also formed one salt-bridge with D306, thus showing R491 along with F506 (that forms one H-bond with N114 of TAK1) play an important role in facilitating the binding of VRK2A and TAK1.This provided important leads toward developing VTMD mutants and study interaction with TAK1 using biochemical and biophysical tools.

**II. Bacterial expression analysis and purification of GST-tagged VRK2A**

1. **Plasmid construction:**
  ➢ VRK2A comprising 1-508 amino acids in pDONR223 vector was obtained from Addgene plasmid repository (Cambridge, MA, USA).
  ➢ The VRK2A DNA of 1532bp size, was further cloned in pGEX-4T vector with an N-terminal GST-tag (Glutathione S-transferase) into BamHI and HindIII restriction sites.
  ➢ All the DNAs were confirmed by restriction digestion and sequencing. Primers used for generating the different constructs have been listed in **Table S2**.
2. **Recombinant bacterial protein expression:**
  ➢ Rosetta pLysS cells (Novagen, Billerica, MA, U.S.A.) were transformed with recombinant pGEX-4T-VRK2A, and colonies were cultured at 37 °C primarily in 5 ml LB media containing ampicillin and chloramphenicol for 18 h as primary culture.
  ➢ Rosetta pLysS cell strains have T7 polymerase which is under the control of Lac operon and are deletion mutants of proteases such as Lon and Outer membrane protease (OmpT). They also carry additional chloramphenicol resistant gene.
  ➢ The primary culture was transferred to two litres flask containing 700 ml pre-warmed media and was further grown at 37 °C with vigorous shaking (180 rpm) to a density of A600 = 0.5.
  ➢ Protein expression was then induced by the addition of 0.1 mM isopropyl-1-thio-D-galactopyranoside (IPTG) and cells were further cultured at 14 °C for 4 to 12 h.
  ➢ Samples of uninduced and IPTG-induced cultures were prepared by adding 6X SDS Laemmli buffer. The 12 h IPTG-induced cells were lysed by sonication, and sampleswere prepared with the centrifuged supernatants containing the proteins. The expression of GST-tagged proteins was analyzed on 12% SDS-PAGE and further confirmed by western blot analysis with anti-GST antibody.
3. **Western blot analysis:**
  ➢ After running the samples on 12% SDS-PAGE, the proteins were transferred to a PVDF (polyvinylidene difluoride) membranes pre-equilibrated with 50 mM Tris-Cl and 150 mM NaCl (TBS buffer).
  ➢ The membranes were further blocked by incubating it in 5% BSA (bovine serum albumin) for 1 h and proceeded for anti-GST (Santacruz, 1:2000 dilution) primary antibodies followed by anti-rabbit (Sigma, 1:10000 dilution) secondary antibody binding.
  ➢ The unbound antibodies were washed with wash buffer (0.1% Tween-20 detergent in TBS buffer).
  ➢ The blot was developed using standard protocols [22] using Super Signal® West Pico Chemiluminescent Substrate Prod by Thermo Scientific.
  ➢ Note: Before loading the protein samples in the 12% SDS-PAGE, the concentration of each of the protein sampleswas estimated using the Bradford assay. Tricolour pre-stained protein ladder was used as standard protein marker.
4. **GST-tagged VRK2A protein purification**
  ➢ For protein purification through GST affinity chromatography, cells expressing the GST-tagged VRK2A were first lysed by sonication (three cycles with 30 s on/off) in pre-chilled lysis buffer containing 20 mM Na2HPO4/NaH2PO4 and 100 mM NaCl at pH 7.0 (PBS buffer) with 0.2% (V/V) Triton X-100, protease inhibitor cocktail in 1:50 dilution (Sigma-Aldrich) and 5mM BME (β mercaptoethanol) [23], [24], [25], [26], [27].
  ➢ BME was added to reduce disulfide bonds as GST tag contains four cysteine residues.
  ➢ Lysates were centrifuged at 22,000 rpm, 4 °C for 30 min in JA-25.50 sorvall rotor (Avanti J-26S XPI, Beckman coulter). The supernatant was incubated with pre-equilibrated (five column volumes) GST-sepharose resin (Novagen, MA, USA) for 1 h at 4 °C.
  ➢ Unbound/weakly bound proteins were washed with five column volume of phosphate buffer saline (PBS buffer)
  ➢ After the stringent washes, GST-tagged VRK2A full length protein was eluted with PBS buffer containing 15 mM reduced glutathione, which was analyzed on 12% SDS-PAGE.
  ➢ The purified GST-VRK2A (84.4 kDa) protein bands were excised and proceeded for identification through mass spectrometry.
5. **Mass spectrometry**
  ➢ The upper three protein bands of VRK2A were excised from the gel and transferred to a microcentrifuge tube.
  ➢ The gels were finely minced, dehydrated in 100 µl of acetonitrile (CH3CN) and centrifuged to remove its extra traces, and then the step was repeated.
  ➢ Gel pieces were reconstituted with 50 µl of 5 ng/µl modified trypsin (Promega) in 50 mM ammonium bicarbonate, incubated on ice for 40 min.
  ➢ After adding 50 mM ammonium bicarbonate, the samples were transferred to 37 °C for overnight incubation.
  ➢ Gel pieces were centrifuged, and the supernatant was saved. Peptides were extracted first with 50 µl of 0.1% formic acid and distilled water, then three times with 50 µl of formic acid and acetonitrile. The mass spectra of the tryptic cleavage products were recorded by Ultraflex II MALDI-TOF/TOF mass spectrometer (Bruker Daltonics).
  ➢ The samples were identified as human VRK2A using peptide mass finger printing by comparing with human SwissProt database using Mascot (Matrix Sciences) search engine.

**Fig. 2:**
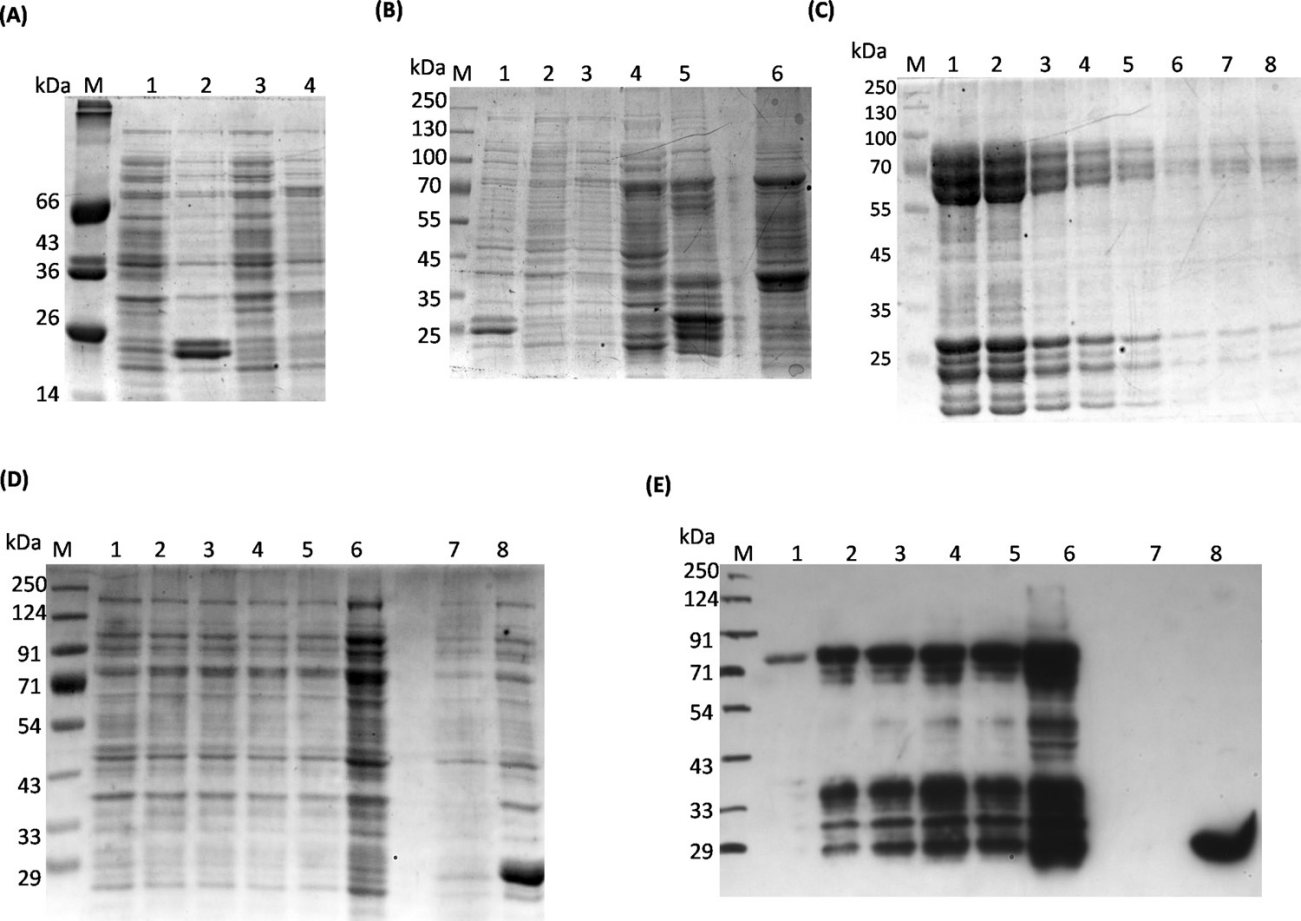
*Expression analysis and purification of GST-tagged VRK2A*. (**A)** Expression of full length GST-tagged VRK2A and GST only proteins. The whole cell proteins after IPTG induction were loaded on 12% SDS-PAGE. Lane M: protein marker; Lane 1 and 2: uninduced and induced GST only protein; Lane 3 and 4: uninduced and induced GST-tagged VRK2A protein (**B)** Solubility analysis of GST-VRK2A proteins through binding the cell lysates to GST beads. The soluble VRK2A protein can be observed in beads bound sample. Lane M: protein marker; Lane 1: induced GST protein; Lane 2 and 3: uninduced and induced GST-VRK2A protein; Lane 4: supernatant after cell lysis; Lane 5: GST beads bound with the protein; Lane 6: cell pellet after lysis **(C)** Multiple bands were observed in elutes (1-8) of purified GST-VRK2A proteins by GST affinity chromatography. **(D)** To compare the change in expression and degradation of GST-VRK2A protein, the whole cell lysate samples were collected at different time point after IPTG induction. **(E)** For better observation, the samples were proceeded for western blot analysis with anti-GST antibody. Lane M: protein marker; Lanes 1 to 5 correspond to whole cell samples before sonication or cell lysis, 1:uninduced; 2 to 5: after 4 h, 6 h, 8 h and 12 h of IPTG induction, respectively; Lane 6: supernatant after cell lysis; Lane 7: bacterial cell lysate as negative control; Lane 8: cell lysate of induced GST only proteins as positive control.

We initially tried expressing and purifying the full-length protein in the bacterial system. The GST-tagged VRK2A was induced with 0.3 mM isopropyl-1-thio-D-galactopyranoside (IPTG); the uninduced and IPTG-induced samples were compared including GST as a control (Fig. 2**A**). The IPTG-induced cells were harvested, lysed and the supernatant was allowed to bind with the GST beads. All the samples were found in soluble forms (Fig. 2**B**). Post-solubility analysis, GST-VRK2A was purified through GST affinity chromatography. As we observed multiple bands of eluted fractions in a 12% SDS PAGE (Fig. 2**C**), the first three lanes of the SDS-PAGE were excised and analysed with the mass spectrometry, which further confirmed its identity (**Fig. S1**). VRK2A stability was determined through expression studies at different time points followed by Western blot analysis, which exhibited degradation of the protein even at 4 h post IPTG induction (Fig. 2**D** and **E**). All these results clearly indicate that the full-length VRK2A protein is highly unstable in bacterial (*E. coli*) system.

**III. Two-step purification of VTMD**

1. **Plasmid construction:**
  ➢ Transmembrane domain of VRK2A or VTMD (comprising 480-508 amino acid residues) was generated by inserting second BamHI site after 479 residue in full length pGEX4T1-VRK2A plasmid using site-directed mutagenesis PCR (polymerase chain reaction)
  ➢ Post-digestion with BamHI, two bands, one of 1443 bp smaller size (BamH1-VRK2A-1-479-BamH1) and of 5056 bp higher size (BamH1-pGEX4T-HindIII-VRK2A-480-508-BamH1) were observed onto 1% agarose gel. The higher size band was gel extracted and self-ligated to generate specific final clone of VTMD domain.
2. **GST-tagged VRK2A-TM (GVTMD) protein purification**
  ➢ GST-tagged transmembrane domain of VRK2A (GVTMD) constructs were expressed in Rosetta pLysS cells as the above-mentioned protocol.
  ➢ Cells expressing the GST-tagged VTMD were lysed by sonication (10 cycles, 30 s on/off) in lysis buffer containing PBS buffer with 0.2% (V/V), protease inhibitor, Triton X-100 and 5 mM BME. Lysates were centrifuged, and the supernatant was incubated with pre-equilibrated GST resin for 1 h at 4°C.
  ➢ Unbound/weakly bound proteins were washed with phosphate buffer saline (PBS buffer) and GVTMD was eluted with buffer containing 15 mM reduced glutathione, which were analyzed on 12% SDS-PAGE.
  ➢ GVTMD (29.7 kDa) was purified by affinity chromatography using GST-sepharose resin (Novagen, MA, USA).
3. **Separation of purified VTMD from GST-tag**
  ➢ The eluted fractions of GVTMD were pooled and extensively dialyzed in PBS (three column volumes).
  ➢ Further, to purify the standalone 3.6 kDa VTMD, the GST-tag was cleaved using thrombin enzyme. A catalytic amount of thrombin (100 NIH units [28]) i.e. 100 μl was added to the 16 ml dialyzed protein solution (total amount of ∼2 mg) and incubated at 20°Cfor 16 h.
  ➢ The solution was passed through the GST column and the flow through (FT) was collected. Thereafter, the elutes were collected after washes with PBS.
  ➢ The protein amount in all the fractions was determined by UV-vis spectrophotometer and Bradford assay. The concentration of the FT containing thrombin and digested VTMD proteins was 0.152 mg/ml (42.5 μM) in 10 ml volume.
    Due to inability in purifying this 3.6 kDa protein through conventional ways, we adopted a two-step centricon-based approach; where these filter units (with specific molecular weight cutoffs) allow lower molecular weight proteins to be filtered-out.
  ➢ First, the cleaved fraction solution was passed through a 10 kDa cutoff centricon, pre-equilibrated with 10 ml PBS buffer to remove the 36 kDa thrombin. Total 8 ml of FT containing cleaved VTMD with 0.06 mg/ml (13.5 μM) concentration was collected.
  ➢ The 3.6kDa VTMD was further concentrated to 24 μM in a 1 kDa cutoff centricon. The fractions were kept for gel-electrophoresis to check purity and size.
4. **Detection of purified VTMD domain**
  ➢ All protein samples including uncut GVTMD, cleaved VTMD, FT and concentrated VTMD were analyzed on 15% SDS-PAGE. While the difference of 3.6 kDa between GVTMD and GST was clearly observed, the small VTMD domain was not visible on 15% SDS-PAGE gels.
  ➢ However, to observe the 3.6kDa VTMD domain, electrophoresis was performed using tricine as trailing ion which has a lower isoelectric point than glycine [29]. 15% Tris-Tricine gels were run at 4°C to reduce protein diffusion/degradation, with anode (2.1M Tris base) and cathode (1M concentration of both Tris and Tricine) in lower and upper chambers, respectively, followed by highly-sensitive silver staining.
  ➢ Silver staining: To observe low molecular weight VTMD, Tris-Tricine gels were stained using silver ammonia solution. The gels were first fixed in 50% methanol for 3 h and washed with autoclaved water thrice for 10 min each. The staining solution was prepared by titrating 48 ml solution A (10M sodium hydroxide and 700μl ammonia solution in autoclaved distilled water) using 2 ml of solution B (20% silver nitrate). The gels were further incubated in staining solution for 20 min on rocker followed by washes with distilled water twice for 15 min each. The gels were then developed by incubating in developer solution (1% citric acid and 38% formaldehyde in autoclaved distilled water). The gels were observed till the bands of proteins appeared. The reaction was stopped by adding destaining solution (40% methanol and 1% acetic acid in distilled water).

**Fig. 3:**
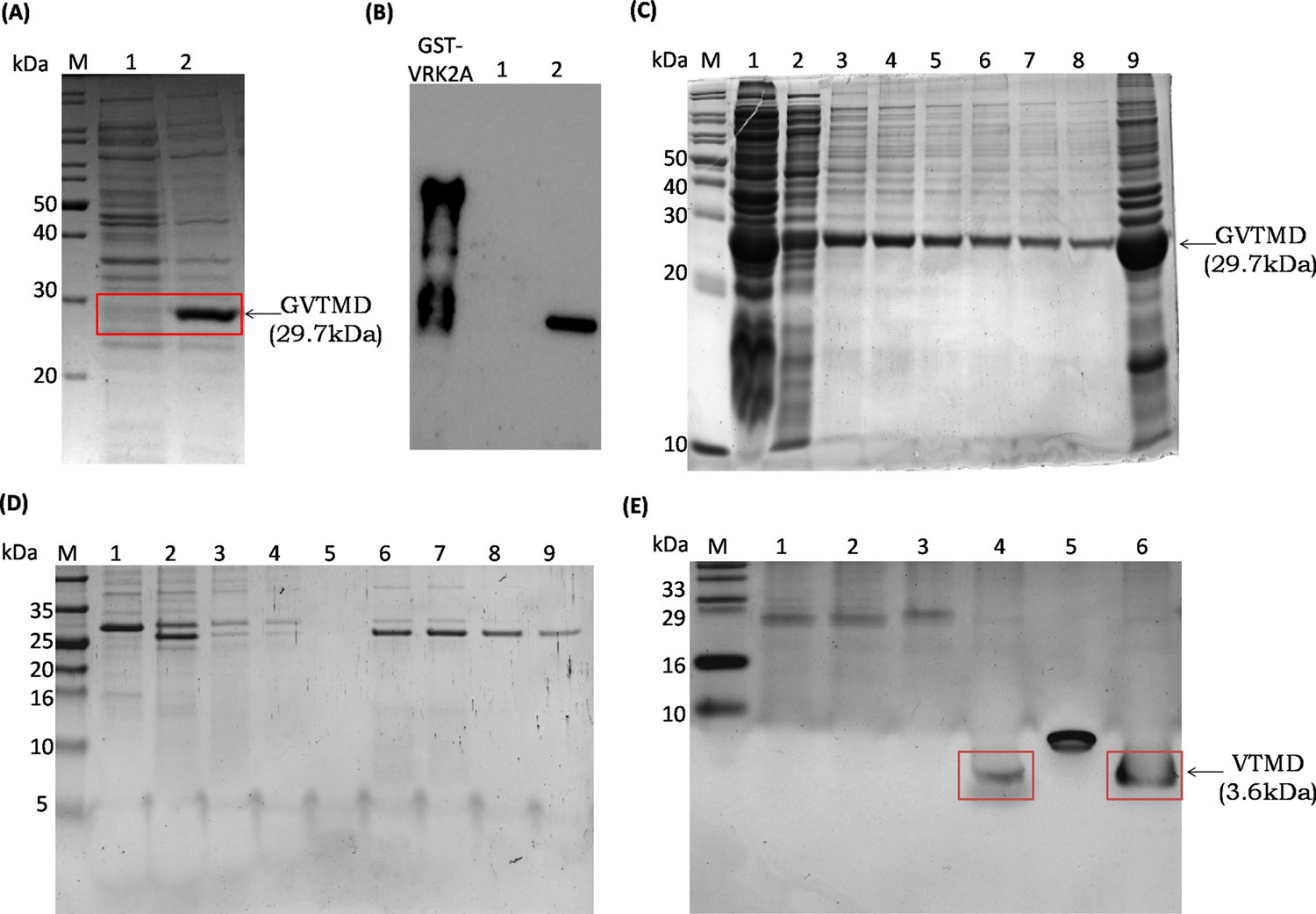
*Expression analysis and two-step purification of VRK2A transmembrane domain (VTMD)*. (**A)** Expression of GVTMD protein was observed in IPTG induced samples on 12% SDS-PAGE gel which was also confirmed with (**B**) western blot analysis using anti-GST antibody and GST-VRK2A lysate as control for the experiment. Lane M: protein marker; Lane 1 and 2: uninduced and induced GVTMD (**C)** The GVTMD protein (29.7 kDa molecular weight) was purified through GST affinity chromatography and observed on 15% SDS-PAGE. Lane M: protein marker; Lane 1: supernatant after cell lysis; Lane 2: flow through; Lane 3 to Lane 8: eluted fractions(E1-E6); Lane 9:cell pellet after lysis.(**D)** After thrombin cleavage of GVTMD protein and binding with GST beads, all the samples were observed on 15% tris-tricine gel.Lane M: protein marker; Lane 1:uncleaved GVTMD; Lane 2:cleaved GVTMD; Lane 3: flow through; Lane 4: first wash; Lane 5: last wash; Lane 6 to Lane 9: eluted fractions (E1-E4). (**E)** The low molecular weight (3.6 kDa) VTMD from the flow through were concentrated and observed in SDS-PAGE after silver staining. Insulin (6 kDa) was used as molecular weight marker. Lane M: protein marker; Lane 1:uncleaved GVTMD; Lane 2: cleaved GVTMD; Lane 3: flow through; Lane 4: concentrated GVTMD; Lane 5: insulin; Lane 6: concentrated GVTMD.

With a cue from the *in silico* studies that highlighted the importance of C-terminal VTMD domain for interaction with TAK1, we sub-cloned it (480–508 residues) in GST-tagged expression vector and proceeded for expression analysis in Rosetta pLysS cells. The expression was observed and confirmed with western blot analysis (Fig. 3**A** and **B**). The GVTMD protein was further purified through GST affinity chromatography, for which the cells were further lysed, centrifuged and the supernatant was allowed to bind with GST beads. After that, the beads were washed and the bound proteins were eluted out with 15 mM reduced glutathione. The collected fractions were found to be more than 95% pure in 12% SDS-PAGE (Fig. 3**C**). To remove the GST-tag from VTMD domain, the pooled eluted fractions of GVTMD were incubated with thrombin enzyme at 20 °C for 16 h. Post thrombin cleavage, the protein fraction was further allowed to bind with GST beads overnight at 4°C in order to remove the cleaved GST fractions. Thereafter the flow through was collected as the protein solution was passed through GST column. The beads were further washed with PBS buffer and the bound GST fractions were eluted with 15 mM reduced glutathione. The samples of GVTMD, the fraction after thrombin cleavage, the flow through, washes and the eluted fractions containing GST were compared on 15% Tris-Tricine gel (Fig. 3**D**). From our result, the molecular weight difference between uncut GVTMD and thrombin digested fraction shows the successful cleavage of GST-tag and purification of 3.6 kDa VTMD domain. To further purify standalone small molecular weight VTMD domain, we used two-step centricon approaches. Firstly, to eliminate higher molecular weight thrombin (36 kDa) present with cleaved fractions of VTMD, the solution was passed through a pre-equilibrated 10 kDa centricon. During this process, our small protein of interest was passed through the column and got collected in the flow through fraction. After cleaving the GST-tag and removing thrombin present in the solution, VTMD domain of higher purity was obtained. The protein was further concentrated using pre-equilibrated 1 kDa centricon. All the samples were analysed on 15% Tris-Tricine gel. Since the small molecular weight protein was not visible through coomassie staining, we stained the gel with silver staining method (Fig. 3**E**). We observed the small standalone VTMD protein of 3.6 kDa molecular weight in the concentrated fractions purified through our novel approach. We successfully purified 3 ml of 0.06 mg/ml standalone VTMD domain from 700 ml *E. coli* bacterial culture. The protein band showed purity around 95% that was sufficient for further biophysical characterizations.

**IV. Thermal stability, tertiary structural characteristics and surface hydrophobicity analysis of GVTMD and VTMD proteins**

1. **Determination of Thermal stability**
  ➢ Thermal stability of GVTMD, VTMD and GST proteins was compared by plotting the far-UV CD data at 208 nm in a temperature range of 20°C–90°C [30].
    Thermal stability of GVTMD, VTMD and GST only proteins (17.5 μM in PBS buffer, pH 7.0) was assessed by monitoring the CD spectrum with increasing temperature. The change in mean residual ellipticity at 208 nm was measured as a function of increasing temperature from 20 to 90°C (Table S3). While GVTMD protein was found to be a stable protein with a melting temperature (T_m_) of ∼64°C, the T_m_ of GST only was found to be 55°C. The T_m_ of untagged VTMD was found to be substantially decreased to 54°C, which is quite typical for a 3.6 kDa protein domain. Overall, the thermal denaturation data demonstrated gain in stability of GST by ∼10°C in presence of the VTMD domain.
2. **Tertiary structure analysis by fluorescence spectroscopy**
  ➢ Fluorescence emission spectra of GVTMD, VTMD and GST only samples in the absence and presence of chemical denaturant 6M urea were measured by a Fluorolog-3 spectrofluorometer (HORIBA Scientific, Edison, NJ, USA) using a quartz cell with 1 mm path-length cuvette at 25°C [31].
  ➢ Emission data of 17.5 μM protein solutions in PBS buffer, pH 7.0 were recorded with 275 nm excitation followed by emission between 300-400 nm. Both the excitation and emission slit widths were kept at 5 nm.

**Fig. 4:**
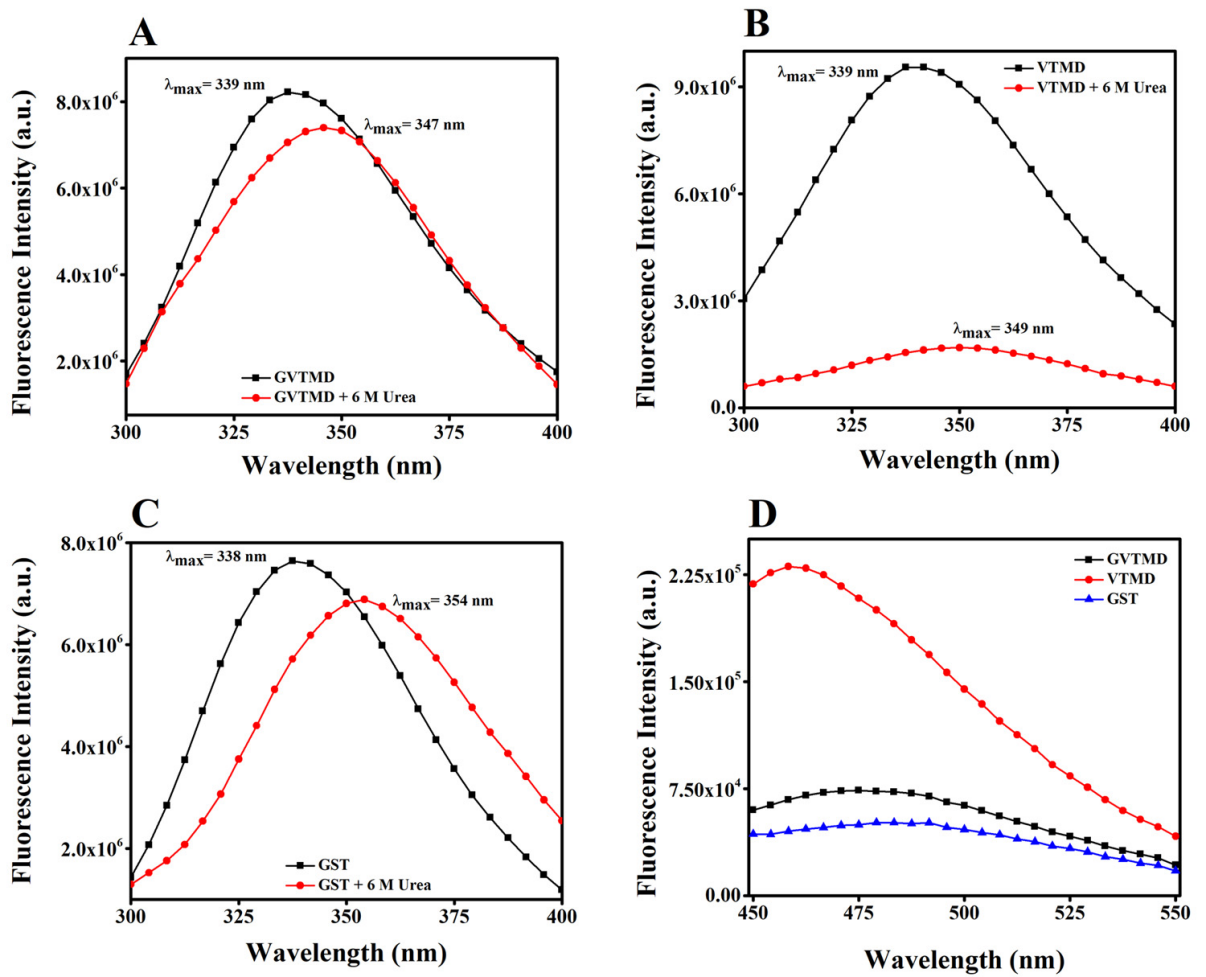
*Intrinsic fluorescence emission spectra and surface hydrophobicity measurements*. Intrinsic fluorescence spectra of **(A)** GVTMD, **(B)** VTMD and **(C)** GST proteins in absence and presence of 6 M urea. Fluorescence spectra of different proteins (17.5 μM in PBS buffer, pH 7.0) were recorded in the range 300–400 nm at 25 °C. The excitation wavelength was 275 nm. Excitation and emissions lit widths were 5 nm each. Data were recorded at a wavelength resolution of 0.5 nm. **(D)** The surface hydrophobicity of GVTMD, VTMD and GST proteins was estimated using a hydrophobic probe ANS at 25 °C. The protein concentration was 0.05 mg/ml and the bis-ANS concentration was 10 μM. The fluorescence spectrum of ANS bound to different samples was recorded in the range 450–550 nm with an excitation wavelength was 390 nm.

To look into the tertiary structural properties of GVTMD and VTMD, the fluorescence emission spectra was measured with an excitation wavelength of 275 nm as the VTMD domain is devoid of any tryptophan residues. In native conditions, the fluorescence emission maxima (λ_max_) for both the GVTMD andVTMD domains were observed at ∼ 339 nm, which indicates a typically well-folded domain. In the presence of 6 M urea, the λ_max_ of both the GVTMD andVTMD along with GST only was red shifted to 347 nm, 349 nm and 354 nm, respectively due to the urea-driven unfolding of the proteins (Fig. 4A, 4B and 4C). The fluorescence intensity of VTMD decreased substantially (∼six fold) in presence of 6 M urea. This decrease in fluorescence intensity along with the red-shift indicates exposure of the fluorophore to a more polar environment under denaturing condition.

3. **Surface hydrophobicity analysis**
  ➢ The surface hydrophobicity of GVTMD, VTMD and GST only was measured with ANS, a specific hydrophobic probe. ANS (10 μM) was added to each of the protein samples [17.5 μM in PBS buffer, pH 7.0] and the mixture was incubated for 60 min at 25°C [32].
  ➢ Fluorescence emission spectra were recorded in the range 450-550 nm using an excitation wavelength of 390 nm. Both the excitation and emission band-passes were 5 nm, respectively. Data were recorded at a wavelength resolution of 0.5 nm.

The surface exposed hydrophobic sites of GVTMD and VTMD were studied using an extrinsic hydrophobic fluorescence probe 8-anilinonaphthalene-1-sulfonic acid (ANS), which binds to protein surfaces via hydrophobic interactions to exposed hydrophobic patches as well as via salt bridge formation between the dye's sulfonate group and positively charged amino acid side chains [33]. The fluorescence intensity of ANS bound to VTMD was increased by ∼70% as compared to GVTMD protein (Fig. 4**D**). A blue shift of ∼17 nm in ANS fluorescence emission maximum, in addition to an intensity increase demonstrated the exposure of hydrophobic surfaces for VTMD contrary to GVTMD. No self-aggregation of VTMD was found when we monitored its absorbance at 400 nm for 3 h (**Fig. S2**). Largely, this data indicates variation in the local environment of VTMD from partially water exposed to fully hydrophobic.

Overall, from the biophysical studies, we identified VTMD as a standalone domain of VRK2A, which is well folded with stability typical of a 3.6 kDa protein.

## Discussion

Purification of small-sized proteins or their domains have always remained a challenge, which multiplies manifold if the protein is a transmembrane domain protein [34]. Using a solubilizing tag such as GST or MBP often complicates the structural and functional analyses due to their huge size and interference in maintaining structural and functional integrity of the domain of interest. In addition to this, removal of the tags often becomes impossible due to various factors [10]. VRK2A is one such protein with a small C-terminal transmembrane domain (3.6 kDa) that regulates important signalling pathways through a wide spectrum of protein-protein interactions. However, inability to purify the full-length protein in the bacterial system remained a major bottleneck in biochemical and biophysical characterizations of these interactions.

It has been observed that the scaffold VRK2A regulates cell stress response by interacting with TAK1, a mitogen-activated protein kinase kinasekinase (MAPKKK), through its C-terminal TM domain in the cytosol [9]. In hypoxic conditions or oxidative stress, the activity of TAK1 protein increases and triggers cascades leading to the activation of c-Jun N-terminal kinase (JNK), which requires interaction with JNK-interacting protein 1 (JIP1) [35]. These interactions are very crucial to modulate MAPK signaling as these proteins stably interact, phosphorylate and form an activation loop. VRK2A interacts with TAK1-JIP1 complex that inhibit their interaction with JNK and its phosphorylation. Unphosphorylated or inactive forms of JNK further inhibit c-Jun phosphorylation, downregulate AP1-dependent transcription and make the cell resistance towards apoptosis.

Therefore, to define the driving force and identify the critical residues of the protein-protein interaction involving VRK2A and TAK1, we attempted expressing full-length VRK2A (1-508) in bacterial expression system harbouring a GST-tag. However, despite several attempts and protocol-modification, the expressed protein was found to be extremely unstable, and degraded while it got expressed as seen on SDS-PAGE as well as through western blot analysis. These membrane proteins are usually difficult to purify due to their low expression level, primary structure, solubility and stability [36,37]. However, there are literature reports on membrane proteins that were purified and further characterized sans reconstitution in micellar medium [38,39]. Since both Tak1 and VRK2A interact in the cytosol, we proceeded with a fast and efficient method to purify the interacting VTMD domain. We subcloned the VTMD domain in the GST tagged expression vector and adopted a unique two-step approach to purify the 3.6 kDa domain alone. Biophysical characterization of the domain showed that it is stable on its own (T_m_∼54°C). We have also dissected the interaction site of VTMD-TAK1 using *in silico* tools.

The important revelations from this study have provided crucial leads toward better understanding of the multi-diverse role of VRK2A transmembrane domain and further characterization of its interaction with TAK1 and other TM-binding proteins. It will also shed light on plausible roles of TM domain of VRK2A in different signaling pathways and effective ways to design small molecules for targeted therapies against associated diseases. This novel approach of purification of a small transmembrane protein domain, visualization and its subsequent characterization will act as a model system for expressing and purifying homogeneous small proteins or domains in the bacterial system for their structural and biophysical characterizations.

## CRediT authorship contribution statement

**Rashmi Puja:** Formal analysis, Visualization, Methodology, Writing – original draft. **Ayon Chakraborty:** Formal analysis, Writing – original draft. **Shubhankar Dutta:** Methodology, Writing – original draft. **Kakoli Bose:** Conceptualization, Visualization, Writing – original draft.

## Declaration of Competing Interest

The authors declare that they have no known competing financial interest or personal relationships that could have appeared to influence the work reported in this paper.

## Appendix Supplementary materials

Image, application 1
