# Supplementary material for: Purification, characterization and functional site prediction of the vaccinia-related kinase 2A small transmembrane domain
Source: MethodsX. 2022 Apr 16;9:101704. doi: 10.1016/j.mex.2022.101704 (PMC9062753; doi:10.1016/j.mex.2022.101704)
Supplement: Supplementary file 1 [file mmc1.docx]

**Supplementary Information**

**Tables**

**Table S1: List of primers used in the study**

| **Primers** | **Sequences** | **T_m_** |
| --- | --- | --- |
| **Forward Primer for VRK2A** | GCCGGATCCATGCCACCAAAAAGA | **57℃** |
| **Reverse Primer for VRK2A** | AAGCTTTCAGAGAAAAAATAAAGCAAGA | **55℃** |
| **Forward primer for BamH1 insertion at 479** | CCCAGTTTACTCTTGGATCCAGTGAAGAGACAAACGC | **58℃** |
| **Reverse primer for BamH1 insertion at 479** | GCGTTTGTCTCTTCACTGGATCCAAGAGTAAACTGGG | **57℃** |
| **pGEX 5’ sequencing primer** | GGGCTGGCAAGCCACGTTTGGTG | **66℃** |

**Table S2: Docking analysis summary of the VRK2A-TAK1 complexes, ranked on the basis of ΔG value**

| **VRK2A-TAK1 complexes** | **Cluster Size** | **Docking Score (ΔG in kcal/mol)** | **RMSD or root mean squared deviation (Å)** |
| --- | --- | --- | --- |
| Cluster 1 | 16822 | -18.2 | 0.9±0.0003 |
| Cluster 4 | 3288 | -13.8 | 0.8±0.009 |
| Cluster 2 | 8980 | -12.7 | 1.6±0.002 |
| Cluster 3 | 3564 | -12.3 | 1.4±0.007 |
| Cluster 5 | 2894 | -10.9 | 1.2±0.0006 |

**Table S3: Thermal melting temperature (T_m_) analysis of GVTMD, VTMD and GST only proteins**

| **Protein Samples** | **T_m_ (°C)** |
| --- | --- |
| **GVTMD** | 64 |
| **VTMD** | 55 |
| **GST only** | 54 |

**Figures**


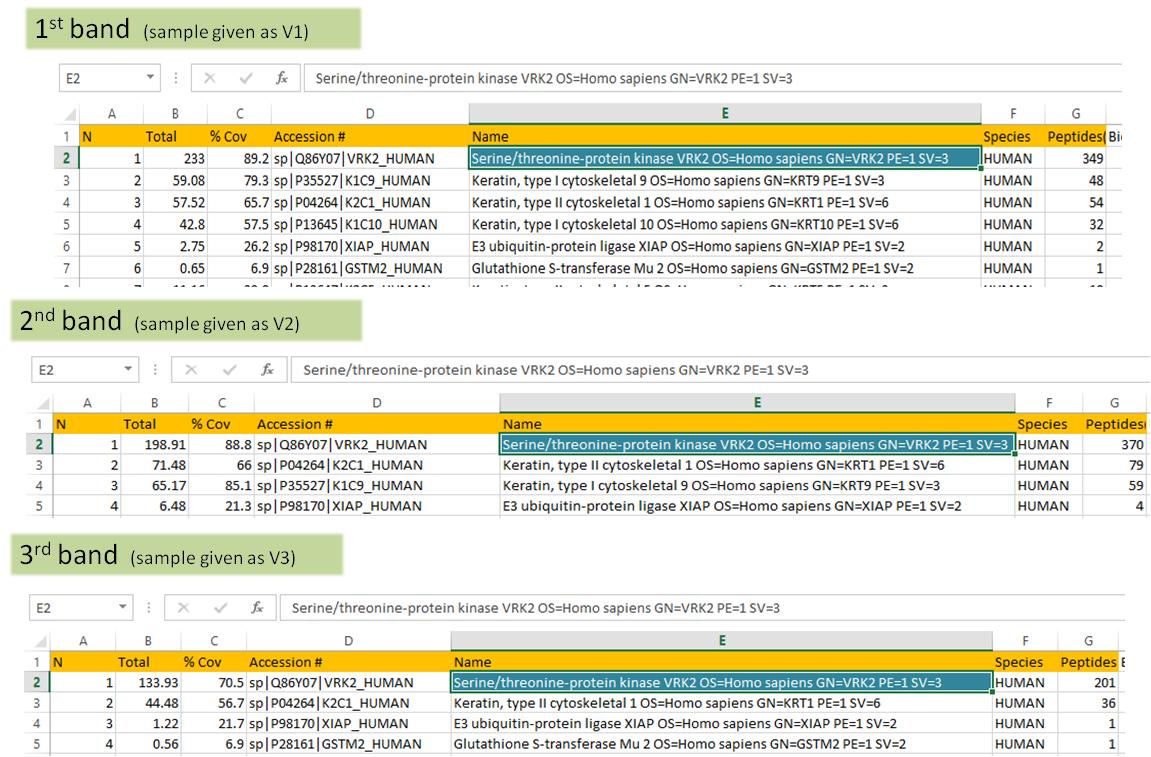


**Figure S1.** Mass spectrometry results analysis of the in-gel digested multiple protein samplesfrom12% SDS-PAGE gel.

**Figure S2. Aggregation profile of VTMD.** The aggregation profile of VTMD protein is checked by monitoring the absorbance of the protein at 400 nm over a period of 3 h.
